# Silk fibroin hydrogel containing *Sesbania sesban* L. extract for rheumatoid arthritis treatment

**DOI:** 10.1080/10717544.2022.2050848

**Published:** 2022-03-11

**Authors:** Duy Toan Pham, Nguyen Thi Phuong Thao, Bui Thi Phuong Thuy, Van De Tran, Thanh Q. C. Nguyen, Ngoc Nha Thao Nguyen

**Affiliations:** a Department of Chemistry, College of Natural Sciences, Can Tho University, Can Tho, Vietnam; b Dong Nai Technology University, Bien Hoa, Dong Nai, Vietnam; c Faculty of Basic Sciences, Van Lang University, Ho Chi Minh City, Vietnam; d Faculty of Pharmacy, Can Tho University of Medicine and Pharmacy, Can Tho, Vietnam

**Keywords:** Silk fibroin, Hydrogel, *Sesbania sesban*, rheumatoid arthritis, anti-inflammation

## Abstract

**Purpose:**

Rheumatoid arthritis, a chronic and progressive inflammation condition in the joints, has significantly reduced the patient quality of life and life expectancy. Crucially, there is no complete therapy for this disease, and the current treatments possess numerous side effects. Thus, novel therapeutic approach is necessary. To that end, this study developed novel silk fibroin in-situ hydrogel containing *Sesbania sesban* L. extract, a plant with high anti-inflammatory actions that are beneficial for rheumatoid arthritis treatments.

**Methods:**

The hydrogels were manufactured using simple method of spontaneous gelation at different temperature. The gel properties of morphology, gelation time, viscosity, gel strength, stability, drug loading capacity, drug release rate, and in-vitro anti-inflammatory activity were investigated with appropriate methods.

**Results:**

The optimal formulation had highly porous structure, with a gelation time of 0.5 h at room temperature and bodily temperature of 37 °C, a viscosity of 2530 ± 50 cP, a gel strength of 1880.14 ± 35.10 g, and a physical stability of >6 months. Moreover, the hydrogel contained the *Sesbania sesban* L. leaf extract with a total phenolic content of 92.8 ± 8.30 mg GAE/g, and sustained the release rate for >20 dạys, followed the Higuchi model. Regarding the in-vitro activities, all formulations were nontoxic to the RAW 264.7 cell line and demonstrated comparable anti-inflammatory activity to the free extract, in terms of the NO reduction levels.

**Conclusion:**

Conclusively, the systems possessed potential properties to be further investigated to become a prospective rheumatoid arthritis treatment.

## Introduction

Rheumatoid arthritis (RA), a chronic and progressive inflammation condition in the joints, is an autoimmune disease with complicated pathology (Guo et al. 2018). This disease mainly affects the synovial joints lining, consequently causes symptoms of arthralgia, redness, swelling, and motion limiting, and ultimately results in the reduced quality of life, disability, and premature death (Guo et al. 2018; Pirmardv & Chegini et al. 2018). Globally, the RA prevalence rate until 2019 was 460 per 100,000 population, with geographically variations (Almutairi et al. 2021). Seriously, although medical treatments have been significantly improved recently, no actual treatment has been proposed for RA (Guo et al. 2018). The current treatments aim to slow down the disease progression to potentially achieve a low disease activity state (Oliveira et al., 2020). These therapies include, but not limited to, non-steroidal anti-inflammatory drugs (NSAID), corticosteroids, and disease-modifying anti-rheumatic drugs (DMARD). Nevertheless, the NSAID and corticosteroids cause numerous side effects (i.e. fluid retention, insomnia, high blood pressure, thin skin) due to a long-term dosing. Furthermore, these medications are only effective in alleviating the joint pain and stiffness, but do not halt the disease progression. DMARD, on the other hand, could alter the RA pathology, consequently delay the disease complications (Grennan et al. 2001). However, despite demonstrating much potentials, these DMARD, both the traditional synthetic drugs (i.e. methotrexate, hydroxychloroquine), biological agents (i.e. TNF-α inhibitor, IL-6 receptor antibody, anti-CD20 antibody), and novel small molecules (i.e. JAK inhibitor), could not completely decrease the long-term RA progression (Guo et al. 2018). Therefore, new therapeutic approaches are necessary.

To this end, hydrogels, crosslinked-network structures of water-soluble polymers possessing similar physiochemical properties to the extracellular matrix, prove their advantages. Hydrogels are biocompatible, versatile in entrapping various therapeutic compounds, possess controlled-release property due to high porosity, and could be easily formulated utilizing mild processing conditions (Li & Mooney 2016; Lee et al. 2018; Vigata et al. 2020; Pham et al. 2021). For RA treatment, local administration of hydrogel to the inflammatory joint sites significantly sustain the drug release rate to more than 20 days and enhance its therapeutic effects compared to the free drug (Kim et al. 2011; Oliveira et al., 2020, 2020). Among numerous hydrogel biomaterials, silk fibroin, commonly extracted from *Bombyx mori* silkworm, has gained increasingly attention due to its biocompatibility, biodegradability, versatility in chemical modification, controlled-release property, among others (Pham et al., 2019, 2020, 2021). Fibroin hydrogels have been proposed and developed for various biomedical applications (Oliveira et al., 2020; Zheng & Zuo 2021; Ziadlou et al. 2021). Nevertheless, although proving much potentials for RA treatment, reports on fibroin-based hydrogels for this purpose are limited. To the best of our knowledge, only one article has been published on this issue (Oliveira et al., 2020). Therefore, it is important to explore this research gap.

Hence, this study presents the development, characterization, and in-vitro anti-inflammatory action of fibroin in-situ hydrogel as a potential drug delivery system for RA treatment. Regarding the drug model to load in the fibroin hydrogel, the extract of sesban (*Sesbania sesban* L. (SS), Egyptian riverhemp, Dien dien (*in Vietnamese*)) has been proposed. SS has been proven, both by ethnopharmacological information and scientific data, to possess several therapeutic activities, especially the anti-inflammatory action that is beneficial for RA treatment (Pandhare et al. 2011; Sajid & PawarVijay 2012; Tatiya et al. 2013; Fitriansyah et al. 2017; Singh et al. 2017; Christman and Gu 2020; Walekhwa et al. 2020; Behl et al. 2021). The SS extract was being determined the total phenolic content at various extraction conditions, and the SS loaded hydrogels were accessed their appearances, sol-gel transition time, viscosity, gel strength, morphology, and stability. Then, the SS release profile in simulated inflammatory condition, in terms of total phenolic content, was evaluated. Finally, the hydrogels cytotoxicity on the macrophage cells RAW 264.7, as well as their anti-inflammatory activity were *in-vitro* investigated.

## Materials and methods

### Materials

The leaves and flowers of SS were collected in Phong Dien district, Can Tho, Vietnam, in April, 2021. Folin-Ciocalteu reagent was imported from Saitong, China. RAW 264.7 cells were bought from ATCC. Dulbecco’s Modified Eagle Medium (DMEM), Fetal Bovine Serum (FBS), PenStrep (penicillin + streptomycin), and trypsin + EDTA were bought from Thermo Fisher Scientific, Singapore. MTS (3-(4,5-dimethylthiazol-2-yl)-5-(3-carboxymethoxy-phenyl)-2-(4-sulfophenyl)-2H-tetrazolium) assay kit was purchased from Promega, USA. Nitric oxide (NO) assay kit was imported from Elabscience, USA. All other chemicals and solvents were of the reagent grades or higher.

### SS extraction and total phenolic content determination

Approximately 1 kg of each plant part, leaves and flowers, was dried under normal temperature, followed by grinding to 1-mm powder. Then, 1 g of the SS leaves/flowers powder was sonication-assisted extracted with 10 mL of EtOH, at a varied temperature of 30, 50, and 60 °C, for a varied extraction time of 60 and 120 min. All SS extractions (SSE) were freshly prepared prior to utilizations.

The SSE total phenolic content, at different extraction conditions, was determined utilizing the Folin-Ciocalteu reagent, following the published method with some modifications (Singleton et al. 1999). Briefly, 0.5 mL of the leaves/flowers SSE was mixed with 2.5 mL of freshly prepared Folin-Ciocalteu solution (10% *v/v*) and 2 mL of Na_2_CO_3_ solution (7.5% *w/v*). The mixture was then incubated at 37 °C in the dark for 1 h, followed by UV-Vis spectroscopic measured at 765 nm (Jasco V-730, Japan). The total polyphenol concentrations were then calculated based on the standard curve with gallic acid (range: 10–50 μg/mL, equation: y = 0.1005x + 0.009, *R*^2^ = 0.9961). The total phenolic content of the extracts was calculated following Equation (1) and expressed as mg gallic acid equivalent (GAE)/g extract on a dry matter basis.

(1) $$\mathrm{Total}\;\mathrm{phenolic}\;\mathrm{content}\;(mg\;\mathrm{GAE}/g)=\;\frac{\mathrm{Amount}\;of\;\mathrm{polyphenol}\;in\;\mathrm{the}\;\mathrm{extract}\;(mg)}{1-\mathrm{The}\;\mathrm{plant}\;\mathrm{powder}\;\mathrm{humidity}}\times 100\%$$

### Fibroin extraction and purification

Fibroin was extracted and purified from the raw *Bombyx mori* silkworm cocoons, following our previous studies (Pham et al., 2018, 2019, 2020). For this, the cocoons were degummed (i.e. sericin removal) using 0.5% (*w/v*) Na_2_CO_3_, washed with de-ionized water, and air dried. Then, the degummed silk (10 g) was cut into small pieces, dissolved in a hot (85 °C) mixture of CaCl_2_:H_2_O:Ca(NO_3_)_2_:EtOH (30:45:5:20 *w/w/w/w*), dialyzed against de-ionized water to remove residual salts, and centrifuged at 10,000 rpm for 30 min. Finally, the fibroin solution was freeze-dried (Heto PowerDry LL3000, Thermo Fisher, USA) at 10^−4 ^Torr and −55 °C. The fibroin powder was stored at −20 °C for further experiments.

### Fibroin hydrogel formulations

Freeze-dried fibroin powder was dissolved in de-ionized water at various concentrations of 1, 2, and 5% (*w/v*). Then, the blank (unloaded) fibroin hydrogel was formulated by allowing 10 mL of the fibroin aqueous solution to spontaneously become solid gel at 4 °C, room temperature (RT, 25 °C), or bodily temperature (37 °C). For the SSE loaded fibroin hydrogel, 10 mL of the SSE, which contained 1 g of the total phenolic content of the SS leaves/flowers, was concentrated to 0.1 mL using the solvent evaporation method. Then, this 0.1-mL SSE was added to 10 mL of fibroin aqueous solution, followed by the incubation at 4 °C, RT, or 37 °C. The SSE loaded fibroin hydrogel was formulated gradually.

### Hydrogel characterizations

The blank and SSE loaded fibroin hydrogels, at a fibroin concentration of 1, 2, and 5%, were characterized in terms of appearance, sol-gel transition time, viscosity, gel strength, gel morphology, and stability.

#### Sol-gel transition time

The sol-gel transition time (gelation time) was determined by the vial inversion method. To this end, 2 mL of the formulations was put in a vial at 4 ± 0.5 °C, 25 ± 0.5 °C (RT), or 37 ± 0.5 °C, the vial was inverted regularly to observe the gel behaviors. The sol- and gel-formation was set as flowing liquid and non-flowing solid, respectively. The sol-gel transition time was determined as the initial time point that the non-flowing solid occurred.

#### Viscosity

The hydrogel viscosity was measured at 25 ± 0.5 °C (RT) by a Brookfield viscometer model DV-E (Brookfield Engineering Labs, USA) utilizing a 40-mm parallel plate configuration with a rotation rate of 15 rpm. The samples (1 mL hydrogels) were placed on the plate and subjected to the viscosity test.

#### Gel strength

The hydrogel strength was determined using a texture analyzer (TA.XT Plus, Stable Micro Systems, UK). Briefly, 1 mL of the blank/SSE loaded fibroin solutions was subjected to a 5-mL vial and incubated at 25 ± 0.5 °C (RT) to form solid gel. The gel was then compressed by a 50-mm hemispherical probe P/0.5 HS to a fixed depth of 4 mm. The gel strength was calculated and demonstrated by the software included in the machine.

#### Morphology

Scanning electron microscopy (SEM, Carl Zeiss Microscopy, 2.00 kV, Germany) was used to observe the hydrogel morphology. The gel, after being fixed, was subjected to a hard resin for ultramicrotome cut into ultra-thin 100-nm slices. These slices were mounted on a metal stub using a sticky carbon disk, followed by sputter coating with gold (10-nm thick). Then, the samples were SEM analyzed.

#### Stability

The hydrogel physical stability was conducted at both RT and 4 °C. At each time intervals (i.e. 1 month, 2 months, 4 months, 6 months), the gels were re-measured their properties of appearance, viscosity, and gel strength. The hydrogels were considered stable when these characteristics remained similar (i.e. in the range of ± 10% of the initial values) to that in the initial time.

### Drug release study

In-vitro cumulative drug release profile of SSE loaded fibroin hydrogel, in terms of total polyphenol, was determined by the shaker method. The hydrogel was designed to be administered directly in the inflamed joint, thus, the drug release study was conducted in a simulated inflammatory joint condition. To this end, 5 mL of SSE loaded fibroin solution (equivalent to 46.4 mg GAE of total polyphenol) were put in a vial to form hydrogel. Then, 2 mL of the PBS buffer, pH 6.5, was added to the hydrogel. The system was continuously shaken at 50 rpm, at 37 °C. At each specific time point, 0.5 mL of samples was withdrawn and buffer refilled. The amount of the total polyphenol content in the samples was determined using the Folin-Ciocalteu reagent, with similar method described in the section “SS extraction and total phenolic content determination”. The concentration of total polyphenol released (µg/mL) at time *t* (*C_t_*) was calculated based on the gallic acid standard curve, and the cumulative drug release percentage was determined followed Equation (2).

(2) $$\%\;\mathrm{Cumulative}\;\mathrm{release}=\frac{C_{t}V_{0}+V\sum_{1}^{t-1}C_{i}}{M_{0}}\;\times \;100\%\;$$

where *V_0_* is the total volume of the release medium (5 mL), *M_0_* is the initial amount of total polyphenol in the hydrogel, *C_i_* is the concentrations of released drug at the time point *i*, and *V* is the withdrawal sample volume at each time point (0.5 mL).

To clarify the drug release mechanism, numerous models including the zero-order, first-order, Higuchi, Hixon-Crowell, and Korsmeyer–Peppas kinetics were tested. The release model possessing the highest *R*^2^ value was considered the most fitted model to describe the drug release behaviors.

### Cell culture

The RAW 264.7 macrophages cell line was cultivated in DMEM supplemented with 10% FBS and 1% PenStrep, at 37 °C and 5% CO_2_. The medium was replaced every other day. The confluent cells (70–80%) were washed, trypsinized using trypsin-EDTA 0.25%, and re-seeded into the 96-well plates for cytotoxicity and anti-inflammatory tests.

### Cytotoxicity test

The cell viability was determined using the MTS assay, with slight modification from a previous study (Pham et al. 2020). To this end, RAW 264.7 cells were seeded into a 96-well plate at a concentration of 5 × 10^3^ cells/well. The cells were then incubated for 24 h at 37 °C and 5% CO_2_, followed by the addition of 20 µL samples at various concentrations of 75, 150, 300, and 500 µg/mL, equivalent to the SSE total phenolic content. The samples included the SSE, the blank fibroin hydrogel, and the SSE loaded fibroin hydrogel. Then, the cells were re-incubated for 24 h at the same condition. Finally, the cells were washed thrice with PBS, and 20 µL of MTS solution was added into each well, followed by incubation for 4 h at 37 °C and 5% CO_2_. The absorbance was measured using UV-Vis plate reader at 490 nm. Mixture of PBS and MTS without cells served as a blank, and the positive control was the untreated cells (i.e. 100% viability). The percentage of cell viability was calculated based on Equation (3).

(3) $$\%\;\mathrm{Viability}=\frac{{\mathrm{Absorbance}}_{\mathrm{sample}}-{\mathrm{Absorbance}}_{\mathrm{blank}}}{{\mathrm{Absorbance}}_{\mathrm{positive}\;\mathrm{control}}-{\mathrm{Absorbance}}_{\mathrm{blank}}}\;\times \;100\%$$

### *In-vitro* anti-inflammatory test

The anti-inflammatory test of the blank fibroin hydrogel, the SSE, and the SSE loaded hydrogel, was conducted using the lipopolysaccharide (LPS) induced RAW 264.7 cells. For this, the cells were seeded in a 96-well plate (3 × 10^4^ cells/well), followed by incubation at 37 °C, 5% CO_2_ for 24 h. Then, the cells were treated with 100 ng/mL of LPS and the samples at concentrations of 75, 150, 300, and 500 µg/mL, equivalent to the SSE total phenolic content. After another 24-h-incubation, the culture media were collected, centrifuged at 3000 rpm for 10 min, and the supernatants were mixed with the NO assay kit following the manufacturer instruction, followed by UV-Vis spectroscopic measured at 550 nm. The NO concentrations were calculated based on the NO calibration curve. The assay negative and positive controls were the non-LPS-induced RAW 264.7 cells and the LPS-induced RAW 264.7 cells without treatments, respectively.

### Statistical analysis

All experiments were conducted in triplicate. Data were expressed in terms of means ± standard deviation (SD). One-way ANOVA and Student’s *t*-test were utilized to determine the significant differences between the samples, with *p* values of <.05 for statistical significance.

## Results and discussions

The long-term RA current treatments of NSAID, corticosteroids, and DMARD, cause lots of side effects and inconveniences for patients. Thus, this study developed and characterized the SSE loaded fibroin in-situ hydrogel for the purpose of both finding the novel therapeutic drug/extract for RA, as well as encapsulating it in a biocompatible, biodegradable, and controlled release hydrogel system to enhance the patient compliance.

**Figure 1.**
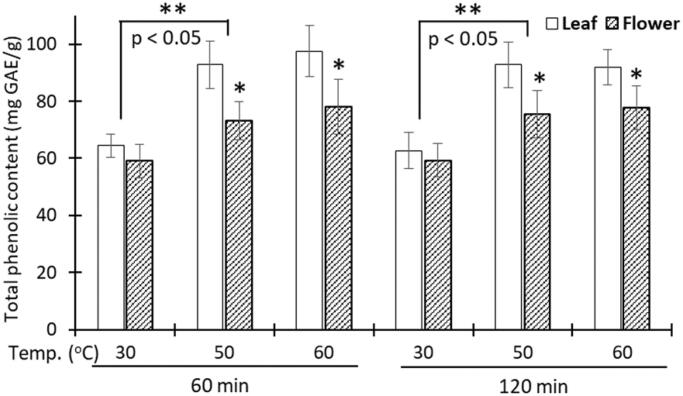
Total phenolic content (mg gallic acid equivalent (GAE)/g) of the *Sesbania sesban* L. leaves and flowers extracts at various conditions of the extraction time of 60 or 120 min, at a temperature of 30 °C, 50 °C, or 60 °C (*n* = 3). *, **: significant differences (*p*<.05) between the flower and leaf extracts, and between the temperature of 30 °C and 50 °C, respectively.

To this end, the SSE was first screened at various extraction conditions to find the optimal preparation with the highest total phenolic content. Phenolic compounds, one of the most diverse groups of plant secondary metabolites, ranging from phenols, flavonoids, to lignins, tannins, and cutins, have been demonstrated to help control the RA condition due to powerful anti-inflammatory properties (Christman & Gu 2020; Behl et al. 2021). Figure 1 demonstrates the total phenolic content, in terms of mg GAE/g, of the SS leaf and flower extracts at 6 different conditions. Obviously, the leaf extract had significantly more phenolic content than the flower one (*p*<.05). Notably, higher temperature of 60 °C and longer extraction time of 120 min did not increase the total phenolic content. Therefore, the best extraction condition was at a temperature of 50 °C and a time of 60 min, which yielded 92.8 ± 8.30 mg GAE/g of the leaf extract. Our result was higher than that of the Indonesian SS leaves, which had a maximum total phenolic content of approximately 51.8 mg GAE/g (Fitriansyah et al. 2017), indicating the effects of geography and nutrition on the plant chemical constituents. Additionally, the SS leaves could possess higher total phenolic content than other species in the genus *Sesbania*. For instance, the total phenolic content of the *Sesbania grandiflora* leaves and flower was about 30 mg GAE/g and 49.1 mg GAE/g, respectively (Munde-Wagh et al. 2012; Shyamalagowri et al. 2010 ).

**Figure 2.**
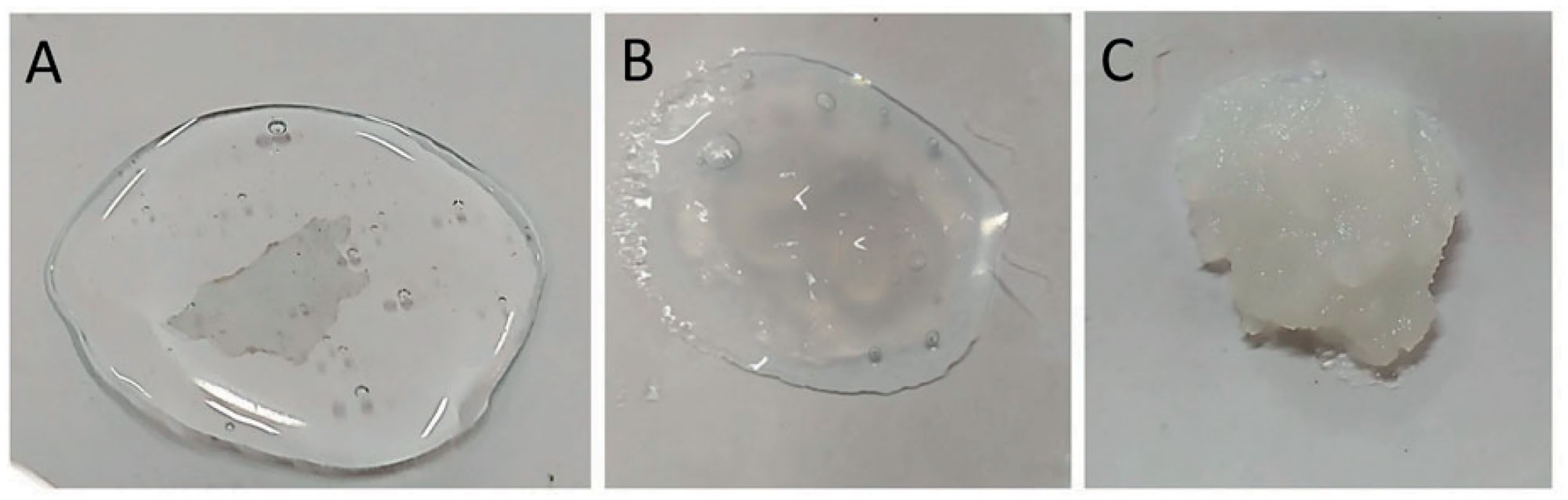
Fibroin hydrogel appearance at a fibroin concentration of (A) 1%, (B) 2%, and (C) 5%.

The best SS leaf extract was then encapsulated in the fibroin hydrogel, followed by gel characterizations. Table 1 shows the properties, in terms of gel appearance, gelation time, viscosity, gel strength, and stability of 6 formulations (3 blank hydrogel and 3 SSE loaded hydrogel) at a fibroin concentrations of 1, 2, and 5%. To this end, an increase of the fibroin amount increases the hydrogel turbidity (Figure 2) and viscosity; and decreases its gelation time at both 4 °C, RT, and 37 °C. These results were consistent with our previous study (Pham et al. 2021), which could be explain due to the transformation of the silk I amorphous water-soluble solution to silk II crystalline water-insoluble gel in the aqueous media. This transformation is affected by the fibroin concentrations, making the hydrogel more solid/viscous and rapidly formed at a high fibroin content (Pham & Tiyaboonchai 2020). Interestingly, the addition of the SSE to the fibroin solution significantly shortened the hydrogel formation time (i.e. from 18 to 2 h at RT for 1% fibroin gel), as well as increased the gel strength. Since EtOH could induce the silk-I-to-silk-II transformation (Pham et al., 2018; Pham & Tiyaboonchai 2020), the SSE, which contained EtOH as the extraction solvent, facilitated this transformation in the fibroin solution, consequently enhanced the gel formation and the gel strength. This fact could be beneficial for the encapsulation of plant extracts in the fibroin hydrogel, as the products are better, in terms of physico-chemical properties, than the blank counterparts. Moreover, a rapid gelation time at 37 °C of the SSE loaded hydrogel makes it suitable for the in-situ sol-gel transition. This offers great benefits for the direct injection of the liquid-form formulation to the inflamed joint, since the liquid would become hydrogel shortly and retains in the joint for an extended time. Lastly, all formulations were physically stable at both 4 °C and RT for at least 6 months, with a viscosity of the SSE loaded hydrogel of 2190 ± 20, 2500 ± 30, and 3010 ± 30 cP, for the fibroin concentration of 1, 2, and 5%, respectively. Similarly, the gel strength was not significantly different than the corresponding hydrogels at the initial time, with a value of 1713.12 ± 18.92, 1930.45 ± 20.74, and 2089.27 ± 32.80 g, for the fibroin concentration of 1, 2, and 5%, respectively. Thus, these data indicated the potentials of the systems. Conclusively, the 2% fibroin formulation was chosen for the next studies.

**Table 1.** Blank and *Sesbania sesban* L. leaf-extract loaded fibroin hydrogel properties (*n* = 3). Results are expressed in terms of mean ± SD. RT: room temperature; I: immediately.

Formulation	Appearance	Gelation time			Viscosity (cP)	Gel strength (g)	Stability	
		4^o^C	RT	37^o^C			4^o^C	RT
1% Fibroin								
Blank	Transparent	>24 h	18 h	12 h	2220 ± 80	1205.15 ± 15.01	>6 Months	
Extract loaded	Transparent	4 h	2 h	1.5 h	2210 ± 50	1693.23 ± 29.83	>6 Months	
2% Fibroin								
Blank	Translucent	>24 h	6 h	4 h	2590 ± 30	1318.02 ± 28.17	>6 Months	
Extract loaded	Translucent	2 h	0.5 h	0.5 h	2530 ± 50	1880.14 ± 35.10	>6 Months	
5% fibroin								
Blank	Turbid	12 h	2 h	1 h	3190 ± 10	1276.71 ± 30.56	>6 Months	
Extract loaded	Turbid	I	I	I	3080 ± 20	2134.38 ± 41.27	>6 Months	

**Figure 3.**
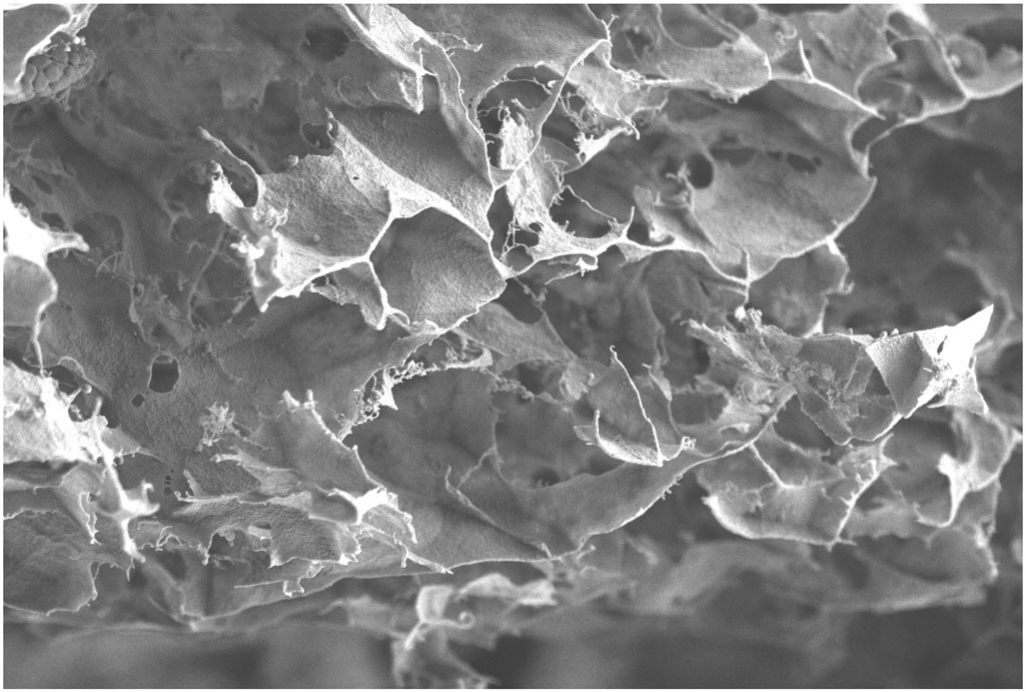
The cross-sectional scanning electron microscopy image of the *Sesbania sesban* L. extract loaded fibroin hydrogel.

**Figure 4.**
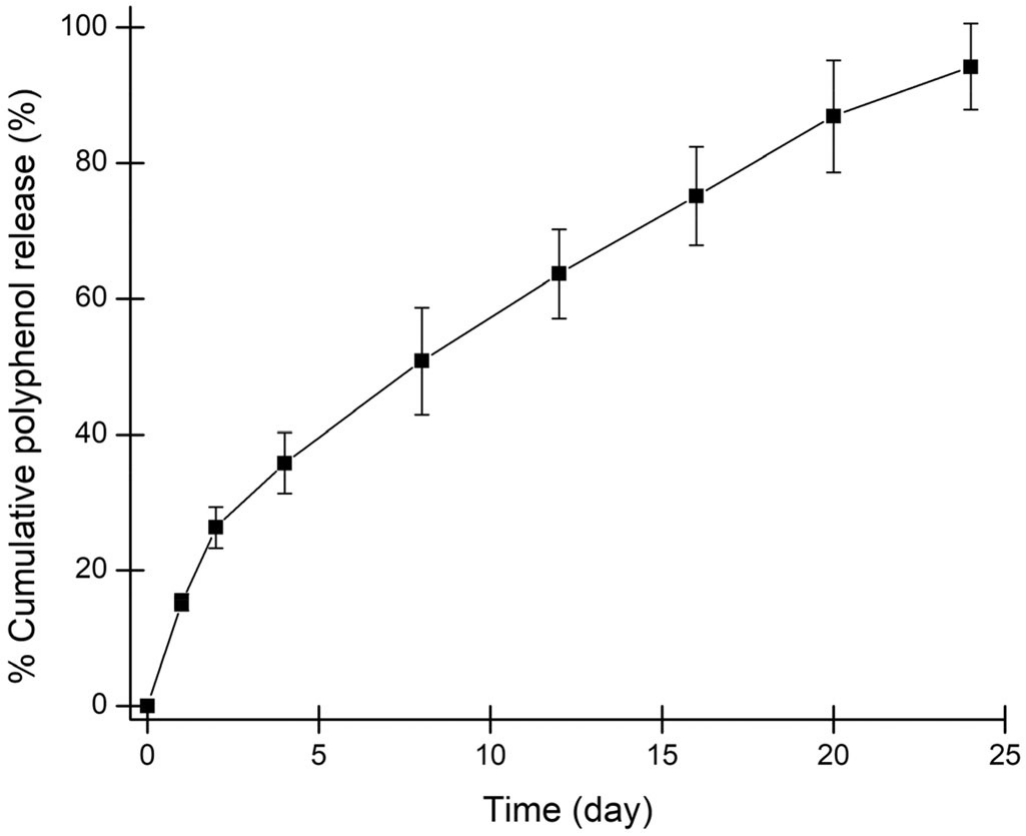
The in-vitro cumulative release profile, in terms of the total phenolic content, of the *Sesbania sesban* L. extract loaded fibroin hydrogel in the simulated inflammatory condition at pH 6.5 (*n* = 3).

The optimal SSE loaded fibroin hydrogel was then characterized its cross-sectional morphology by SEM (Figure 3) and in-vitro release profile in the inflammation condition at pH 6.5 (Figure 4). The hydrogel illustrated a highly porous structure that increases the system surface areas, making the drug/extract has more interactions with the fibroin. Consequently, these interactions potentially sustained the release rate of the SSE, in terms of total phenolic content, to more than 20 days. Moreover, mathematical curve fitting of the release profile to various models of zero order (*R*^2^ = 0.9500), first order (*R*^2^ = 0.5284), Higuchi (*R*^2^ = 0.9973), Hixon-Crowell (*R*^2^ = 0.6116), and Korsmeyer–Peppas (*R*^2^ = 0.6842), showed that the most fitted one was Higuchi model, with an equation of y = 19.488x − 2.3553. This indicates that the release profile is based on diffusion-controlled mechanism, corresponding to the gradual biodegradability of fibroin in the medium (Pham et al. 2021). Since the hydrogel was designed to be administered by directly injection of the gel-in-solution-state to the joint, followed by the spontaneously gel formation, these results are beneficial for the RA patient compliance since one therapeutic dose might last for one month, without the need for re-administration.

**Figure 5.**
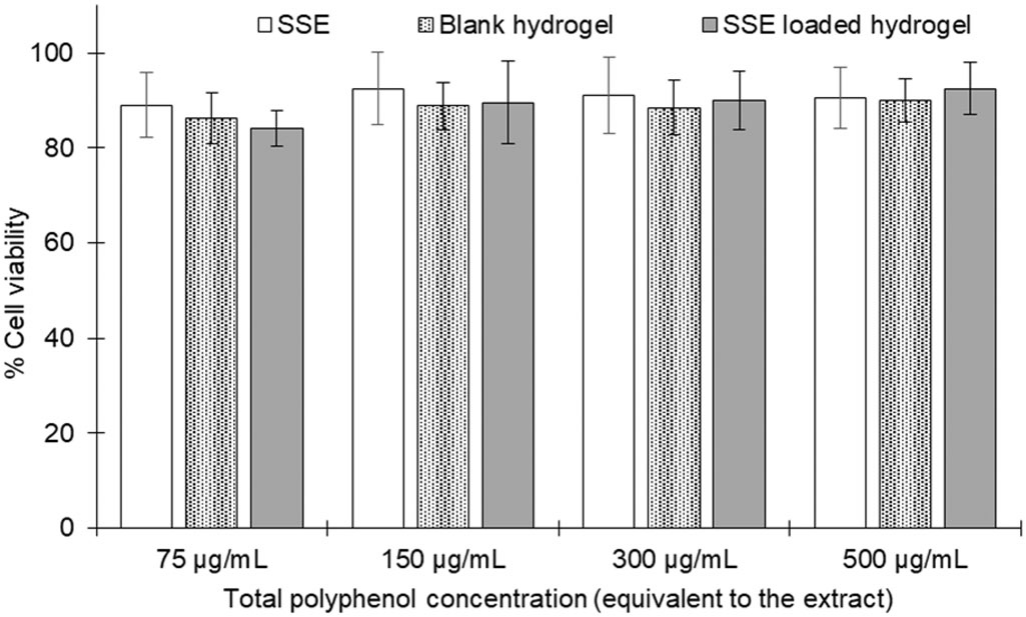
Cytotoxicity MTS assay on the RAW 264.7 cell line of the *Sesbania sesban* L. extract (SSE), the blank (unloaded) fibroin hydrogel, and the SSE loaded fibroin hydrogel at the concentrations of 75, 150, 300, and 500 µg/mL, equivalent to the total phenolic content (*n* = 3).

**Figure 6.**
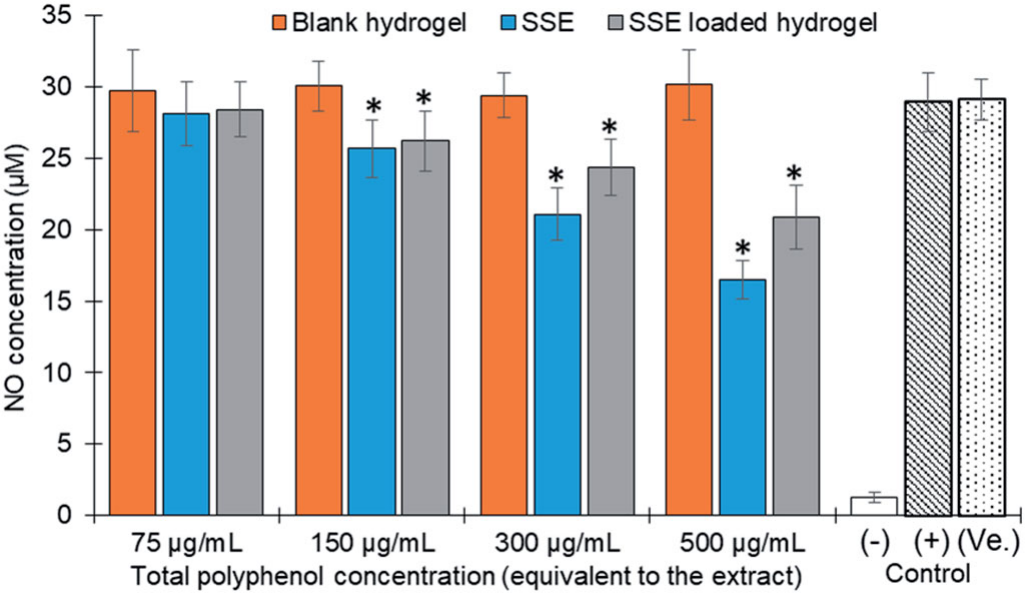
Anti-inflammatory activities, in terms of the NO concentration reduction, on the LPS-induced RAW 264.7 cell line of the *Sesbania sesban* L. extract (SSE), the blank (unloaded) fibroin hydrogel, and the SSE loaded fibroin hydrogel at the concentrations of 75, 150, 300, and 500 µg/mL, equivalent to the total phenolic content (*n* = 3). The negative (−) and positive (+) were the non-LPS-induced RAW 264.7 cells and the LPS-induced RAW 264.7 cells without treatments, respectively. *: significant differences (*p*<.05) between the SSE/SSE loaded hydrogel and the blank hydrogel; (Ve.): vehicle control.

Regarding the SSE loaded fibroin hydrogel therapeutic activities on the RA treatment, the cytotoxicity assay and the anti-inflammatory test on the macrophage cell line RAW 264.7 were conducted. To this end, all SSE, blank fibroin hydrogel, and the SSE loaded fibroin hydrogel, at a concentration range from 75 to 500 µg/mL (equivalent to the total phenolic content) possessed no potential cytotoxicity to the RAW 264.7 cells, with a cell viability of >80% (Figure 5). This suggests that the formulations were safe to the cells and are suitable for the anti-inflammatory test. Interestingly, the *in-vitro* anti-inflammatory assay demonstrates that both the SSE and the SSE loaded fibroin hydrogel possessed better activity, with lower NO concentration, than the blank hydrogel (Figure 6). Furthermore, no significant difference was noted between the SSE in the free form and in the hydrogel, indicating the effects of the hydrogel for reducing the inflammation in the RA disease.

## Conclusions

This study presents the novel silk fibroin in-situ hydrogel containing *Sesbania sesban* L. extract for rheumatoid arthritis treatment. The optimal hydrogel contained the plant leaf extract with a total phenolic content of 92.8 ± 8.30 mg GAE/g. Physico-chemically, the gel was translucent with a highly porous structure, possessed a gelation time of 0.5 h at bodily temperature of 37 °C, a viscosity of 2530 ± 50 cP, a gel strength of 1880.14 ± 35.10 g, and a physical stability of >6 months. The system could sustain release the extract for >20 dạys, followed the Higuchi model. Furthermore, all formulations were nontoxic to the RAW 264.7 cell line and demonstrated comparable anti-inflammatory activity to the free extract. These data, together with the inherent RA treatment activity of the phenolic compounds, make the product a potential system to be a novel treatment for RA in the future.

## Data Availability

The data that support the findings of this study are available from the corresponding authors, Duy Toan Pham and Bui Thi Phuong Thuy, upon reasonable request.
